# The Vacuolar ATPase from *Entamoeba histolytica*: Molecular cloning of the gene encoding for the B subunit and subcellular localization of the protein

**DOI:** 10.1186/1471-2180-8-235

**Published:** 2008-12-23

**Authors:** Mayra Gisela Meléndez-Hernández, María Luisa Labra Barrios, Esther Orozco, Juan Pedro Luna-Arias

**Affiliations:** 1 Departmento de Infectómica y Patogénesis Molecular, Cinvestav-IPN, Av. IPN 2508, Col. Zacatenco, CP07360, D. F. México, México; 2 Departmento de Biología Celular, Cinvestav-IPN, Av. IPN 2508, Col. Zacatenco, CP07360, D. F. México, México

## Abstract

**Background:**

*Entamoeba histolytica *is a professional phagocytic cell where the vacuolar ATPase plays a key role. This enzyme is a multisubunit complex that regulates pH in many subcellular compartments, even in those that are not measurably acidic. It participates in a wide variety of cellular processes such as endocytosis, intracellular transport and membrane fusion. The presence of a vacuolar type H^+^-ATPase in *E. histolytica *trophozoites has been inferred previously from inhibition assays of its activity, the isolation of the *Ehvma1 *and *Ehvma3 *genes, and by proteomic analysis of purified phagosomes.

**Results:**

We report the isolation and characterization of the *Ehvma2 *gene, which encodes for the subunit B of the vacuolar ATPase. This polypeptide is a 55.3 kDa highly conserved protein with 34 to 80% identity to orthologous proteins from other species. Particularly, *in silico *studies showed that EhV-ATPase subunit B displays 78% identity and 90% similarity to its *Dictyostelium *ortholog. A 462 bp DNA fragment of the *Ehvma2 *gene was expressed in bacteria and recombinant polypeptide was used to raise mouse polyclonal antibodies. EhV-ATPase subunit B antibodies detected a 55 kDa band in whole cell extracts and in an enriched fraction of DNA-containing organelles named EhkOs. The V-ATPase subunit B was located by immunofluorescence and confocal microscopy in many vesicles, in phagosomes, plasma membrane and in EhkOs. We also identified the genes encoding for the majority of the V-ATPase subunits in the *E. histolytica *genome, and proposed a putative model for this proton pump.

**Conclusion:**

We have isolated the *Ehvma2 *gene which encodes for the V-ATPase subunit B from the *E. histolytica *clone A. This gene has a 154 bp intron and encodes for a highly conserved polypeptide. Specific antibodies localized EhV-ATPase subunit B in many vesicles, phagosomes, plasma membrane and in EhkOs. Most of the orthologous genes encoding for the EhV-ATPase subunits were found in the *E. histolytica *genome, indicating the conserved nature of V-ATPase in this parasite.

## Background

*Entamoeba histolytica *is the protozoan parasite which causes human amebiasis. It is estimated that between 40,000 and 100,000 people die annually worldwide from this condition [1]. Four sequential steps have been described during the trophozoite-target cell interaction: 1) adherence, 2) extracellular cytolysis, 3) contact-dependent cytolysis and 4) phagocytosis [2]. Lysis of epithelial cells inside trophozoites requires specific and precise pH that is provided in different vacuoles [2]. The vacuolar H^+^-ATPase (V-ATPase) is the key enzyme in many, if not all, acidification processes inside vacuoles. This enzyme is a multisubunit complex that translocates protons across membranes against their electrochemical potential through ATP hydrolysis. The V-ATPase is formed by the V_0 _complex, corresponding to the integral membrane sector, and the V_1 _complex that constitutes the globular headpiece responsible for the catalytic activity [3-5]. The V-ATPase is located in endoplasmic reticulum, secretory vesicles, Golgi vesicles, clathrin-coated vesicles, endosomes, lysosomes, storage vesicles, synaptic vesicles and the central vacuole (in plants and fungi), but it can also be found in plasma membranes [3,4]. V-ATPase also participates in the biosynthetic and endocytic pathways, transmembrane transport of viral contents and toxins, and in coupled transport of small molecules [3-6]. Moreover, V-ATPase is involved in cytosolic pH regulation, in Na^+^, Ca^2+ ^and Cd^2+ ^uptake *via *H^+^-driven antiport, in H^+^-dependent transport of monoamines and γ-aminobutyrate neurotransmitters carried out by the difference in H^+ ^concentration, and in glutamate uptake driven by the membrane voltage [3-6]. Additionally, it is thought that the V-ATPase is the pH sensor that regulates transport from early to late endosomes. This assumption is supported by the interaction between V-ATPase and the small GTP-binding protein ARF6 and its GDP/GTP exchange factor ARNO in a pH-dependent manner [7].

Several years ago acidification inhibition experiments of pinocytic vesicles with bafilomycin A1 revealed the presence of the vacuolar ATPase in *E. histolytica *[8]. However, only two genes encoding for *E. histolytica *ATPase subunits have been cloned: *Ehvma1 *is an intron-less gene that encodes for the 67 kDa subunit A of V_1 _complex [9]. *Ehvma3 *encodes for an 18.1 kDa polypeptide corresponding to the c subunit of the V_0 _complex [10]. Recently, proteins related to V-ATPase have been identified by proteomic analysis of purified phagosomes in *E. histolytica *[11,12]. In order to continue with the study of subunits forming the ATPase in this parasite and to investigate their role in phagocytosis, we report here the cloning and characterization of the *Ehvma2 *gene which encodes for the *E. histolytica *B subunit of the V_1 _complex. We also performed the subcellular location of its encoded protein in trophozoites during phagocytosis.

## Results

### Cloning and characterization of the gene encoding for the subunit B of the vacuolar ATPase of *E. histolytica*

**Figure 1:**
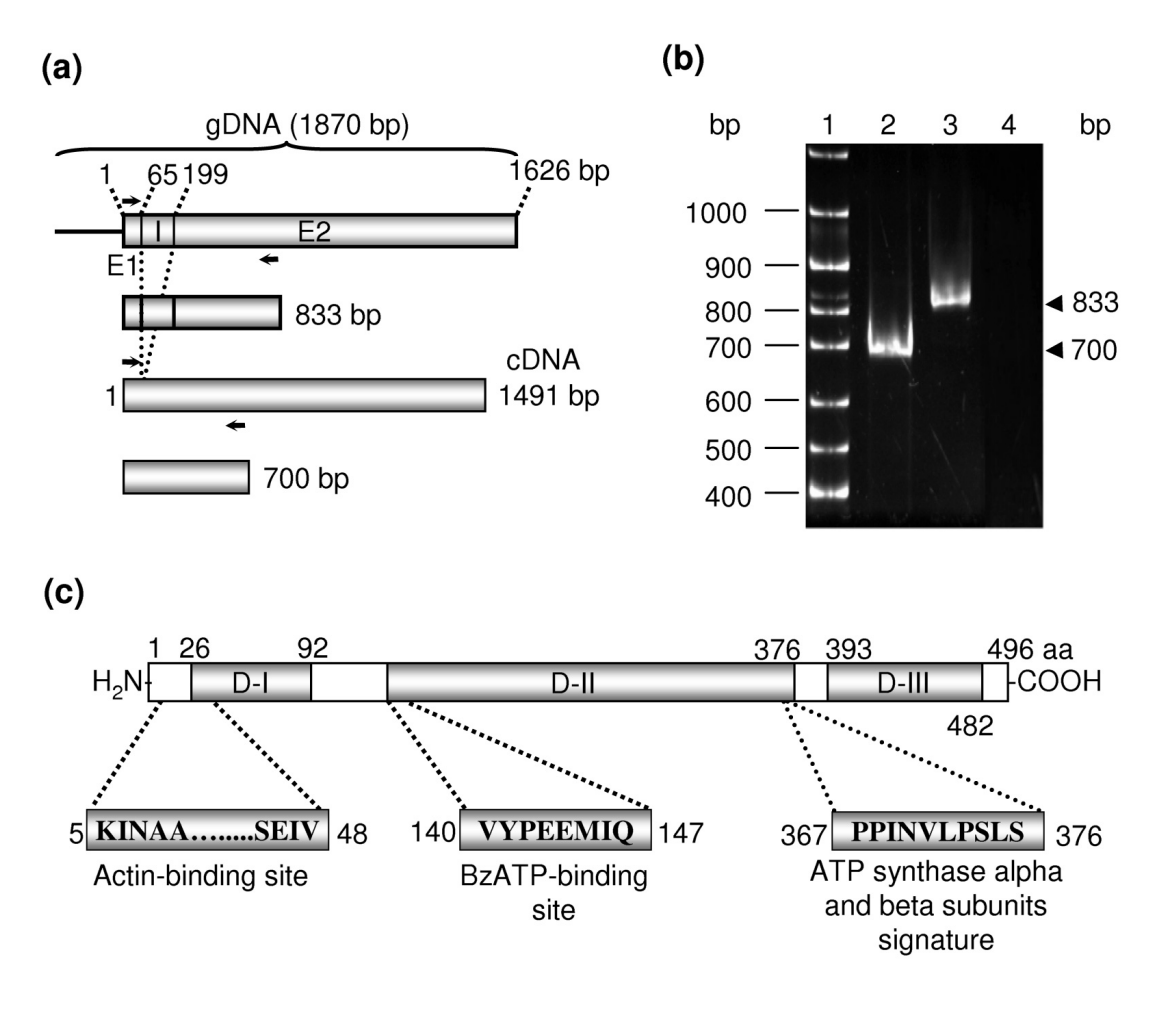
**Organization of the *Ehvma2 *gene**. (a) *Ehvma2 *gene is structured by exons E1 (1–64 nt) and E2 (200–1,626 nt) separated by an intron (I) of 135 bp (65–199 nt). Arrows show position Bvac-S-1 (sense) and Bvac-AS-833 (antisense) primers used in PCR with genomic DNA (gDNA) and RT-PCR. Predicted lengths of DNA amplified from gDNA (833 bp) and from cDNA (700 bp) are also shown. (b) EtBr stained 4% PAGE gel. Lane 1, 100 bp DNA ladder; lane 2, DNA amplified by RT-PCR; lane 3, DNA amplified with gDNA; lane 4, negative control of RT-PCR where no reverse transcriptase was used. (c) Structure of EhV-ATPaseB showing the beta-barrel domain (D-I), the nucleotide-binding domain (D-II), the C-terminal domain found in the ATP synthase alpha/beta family (D-III), the putative actin-binding site, the BzATP-binding site sequence, and the signature of ATP synthase alpha and beta subunits.

A 1,870 bp DNA fragment (amplified using S-Bvac and AS-Bvac primers) was cloned into the pGEM-T-Easy vector. DNA sequencing revealed that cloned DNA contains two open reading frames (ORFs) of 65 (E1, 1–64 nt) and 1,427 bp (E2, 200–1626 nt), separated by a 135 bp non-coding region (I, 65–199 nt) (Fig. 1a). In region I we localized a splicing consensus sequence for nuclear-encoded genes, suggesting that it could be an intron. RT-PCR assays using the primers Bvac-S-1 and Bvac-AS-833 gave a 700 bp product, whereas amplification with genomic DNA produced an 833 bp fragment, indicating that *Ehvma2 *gene (UniProtKB/TrEMBL entry Q4VSM4) contains a 135 bp intron (Fig. 1a, b). *Ehvma2 *gene from clone A was 100% identical to the corresponding gene of *E. histolytica *HM1:IMSS (locus EHI_189850) reported in the *E. histolytica *genome data bank. It encodes for a predicted 496 amino acid polypeptide of 55.3 kDa and a pI of 4.9. Blast search of the amino acid sequence encoded by *Ehvma2 *revealed a high degree of conservation with other V-ATPase B subunits from a number of organisms with an identity from 34% to 78% and a similarity varying from 53% to 90%.

The EhV-ATPase B subunit (EhV-ATPaseB) has three domains (Fig. 1c): Domain I, (aa 26–92) is the beta-barrel domain (Pfam domain PF02874). Domain II (aa 140–376) corresponds to the core region containing the highly conserved nucleotide binding domain (PF00006). Domain III (aa 393–482) is the domain found in the ATP synthase alpha/beta family (PF00306). The conserved central domain contains the P-loop structure, which forms a flexible loop that interacts with one of the phosphate groups of ATP in α and β subunits of F-ATPase, and in V-ATPase subunit A [13]. V-ATPase B subunit from *Bos taurus *brain cells has a region which works like a P-loop and binds BzATP, an ATP analog [14]. B subunits conserve all residues implicated in the interaction with actin, the nucleotide binding site and the ATP synthase alpha and beta subunits signature. The actin-interaction domain found in EhV-ATPase B subunit was located in the region from K5 to V48, the BzATP consensus binding site ([IV]-Y-P- [EQ]-E-M-I- [QES]) was localized from V140 to Q147 and the ATP synthase alpha and beta subunits signature (P- [SAP]- [LIV]- [DNH]-xxx-S-x-S) was found in the region from P367 to S376 (Fig. 1c). Finally, the EhV-ATPaseB N- and C-terminal regions differed in size and sequence with respect to the corresponding ends of B subunits from other organisms.

### Expression of a specific polypeptide of the *E. histolytica *vacuolar ATPase subunit B in bacteria

**Figure 2:**
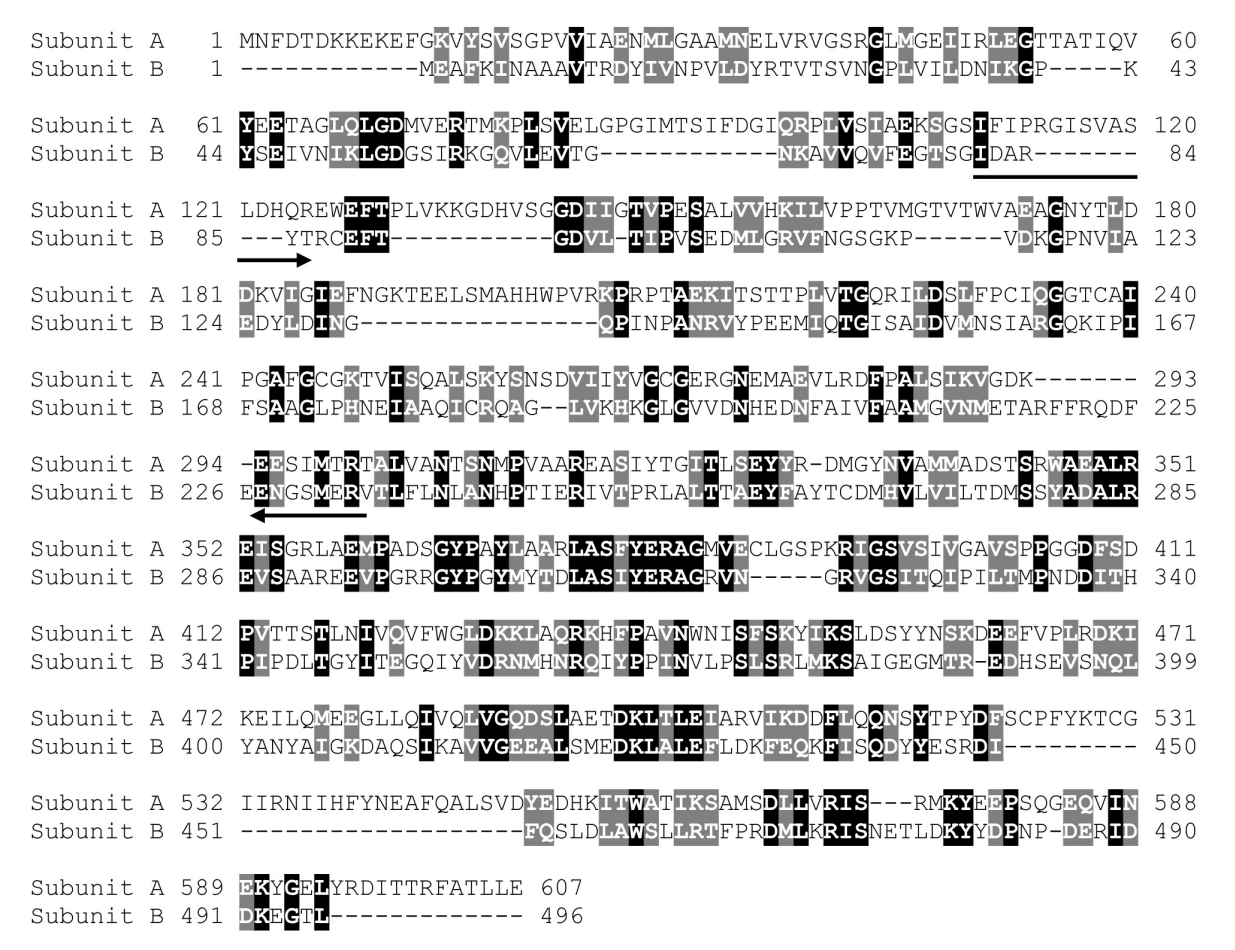
**Alignment of polypeptide sequences of subunits A and B of the EhV-ATPase**. Alignment was performed using the ClustalW program. Identical amino acids are shown in black boxes. Conserved changes are shown in gray boxes. Numbers correspond to amino acid positions in each polypeptide. Arrows indicate primers Bvac-S-373 and Bvac-AS-833 used to amplify by PCR a 465 bp DNA fragment containing a 154 aa region, which is a EhV-ATPaseB specific region.

**Figure 3:**
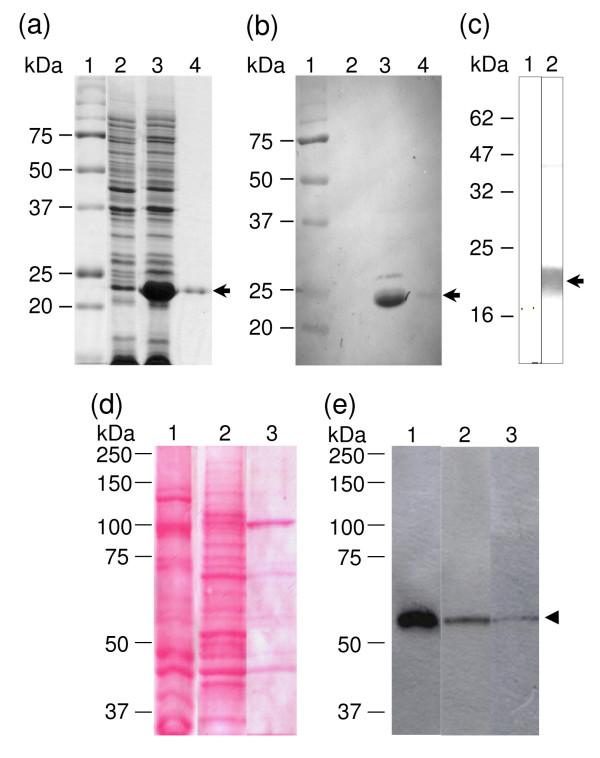
**Expression of a recombinant 21 kDa polypeptide (rEhBvac21) from the EhV-ATPase B subunit and immunodetection of the endogenous protein by Western blot assays**. rEhBvac21 polypeptide was IPTG induced in bacteria and purified through a Ni^2+^-NTA-agarose column as described in Methods. (a) Coomassie Blue stained 15% SDS-PAGE gel. Lane 1, protein molecular weight markers; lane 2, non-induced bacteria; lane 3, induced bacteria; lane 4, purified rEhBvac21 polypeptide. (b) Western blot assay of gel shown in (a), using anti-6His-tag monoclonal antibodies and horseradish peroxidase-conjugated anti-mouse IgG secondary antibodies (1:1,000), and revealed as described. (c) Western blot of purified rEhBvac21 polypeptide using: lane 1, mouse pre-immune serum or lane 2, mouse polyclonal anti-rEhBvac21 antibodies (1:1,000), and horseradish peroxidase-conjugated anti-mouse IgG secondary antibodies (1:1,000). (d) Ponceau stained nitrocellulose membrane of proteins (see Methods) separated by 10% SDS-PAGE. Lane 1, whole trophozoites; lane 2, soluble fraction; lane 3, EhkO-enriched fractions. (e) Immunodetection of proteins shown in (d), with mouse polyclonal anti-rEhBVac21 antibodies (1:20,000) and horseradish peroxidase-conjugated goat anti-mouse IgG secondary antibodies (1:3,000) using the ECL Plus detection kit as described. Arrow shows the rEhBvac21 polypeptide. Arrow-head indicates the endogenous V-ATPse B subunit.

To determine if the *E. histolytica *genome contains sequences related to the V-ATPase B subunit, we performed a BLAST search and found that the V-ATPase A subunit was the only sequence related. It showed 23% identity and 38% similarity to EhV-ATPaseB (Fig. 2). The most divergent region between these sequences resides in their N-terminal ends. Thus, we selected a 462 bp region (I81 to V234) of the gene encoding for the V-ATPase subunit B to be cloned in frame into pRSET A vector to express a 21 kDa recombinant His-tagged polypeptide (rEhBvac21) in *E. coli *(Fig. 3a, lane 3). This polypeptide was purified by immobilized metal affinity chromatography (IMAC) through a Ni^2+^-NTA agarose column under denaturing conditions (Fig. 3a, lane 4). In Western blot assays the anti-6His-Tag monoclonal antibodies recognized the induced rEhBvac21 polypeptide band in extracts of 1 mM IPTG induced bacteria (Fig. 3b, lane 3) and purified rEhBvac21 (Fig. 3b, lane 4). This result confirms the nature of induced polypeptide. Therefore, rEhBvac21 protein was electroeluted from 12% SDS-PAGE gels and used to produce mouse polyclonal anti-rEhBvac21 antibodies that specifically identified rEhBvac21 in induced bacteria (Fig. 3c, lane 2), while pre-immune serum did not recognize any bacterial polypeptide (Fig. 3c, lane 1). Mouse polyclonal anti-rEhBvac21 antibodies reacted with a 55 kDa band in trophozoite total extracts (Fig. 3d, e, lanes 1), which corresponds to the predicted molecular weight of the full length EhV-ATPase B subunit.

### Localization of the subunit B of V-ATPase in *E. histolytica *trophozoites

**Figure 4:**
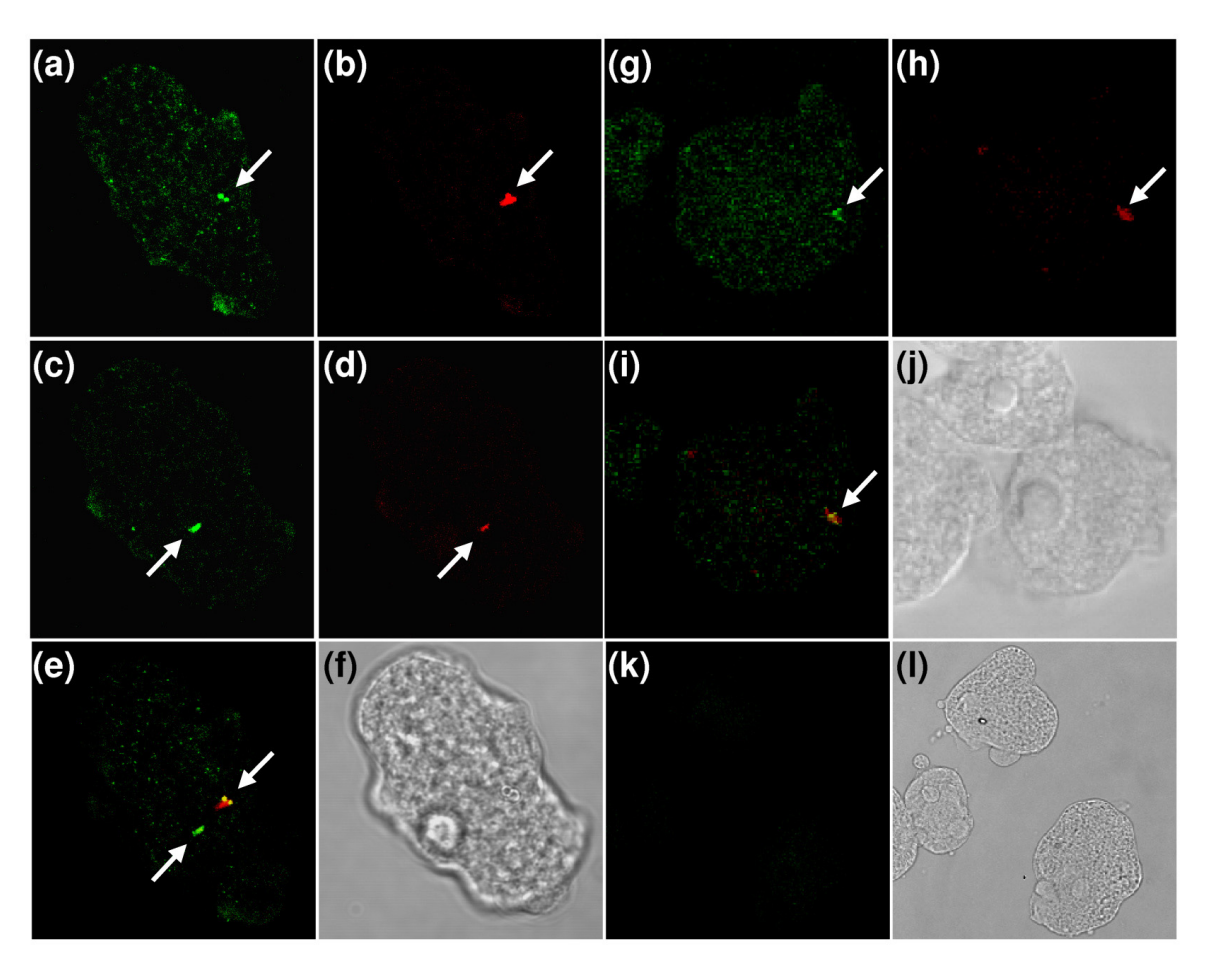
**The EhV-ATPase B subunit colocalized with the DNA-containing organelles (EhkOs)**. Trophozoites were fixed, permeabilized and incubated with anti-rEhBvac21 antibodies. Then, they were incubated with FITC-labeled goat anti-mouse antibodies and observed through a laser confocal microscope. (a, c) Immunodetection of EhV-ATPase B found within two different planes of the same cell (green channel). (b, d) Sections observed in (a) and (c), respectively, showing the DNA staining (red channel). (e) Merge of green and red fluorescence emissions shown in (a) through (d) which correspond to the two cellular planes mentioned before. (f) Cell from panels (a)-(e) observed by light microscopy. (g) EhV-ATPaseB localization in a section of another cell observed in green channel. (h) Same plane as (g), but observed in red channel. (i) Merge of fluorescence signals emitted in (g) and (h). (j) Cells of panels (g)-(i) observed by light microscopy. (k) Cells incubated only with secondary antibodies as a negative control and observed in green channel (maximal projection). (l) Cells of negative control observed by light microscopy. Arrows indicate EhkOs.

We carried out immunodetection experiments of endogenous EhV-ATPaseB protein using anti-rEhBvac21 antibodies and paraformaldehyde-fixed trophozoites. Through confocal microscopy, antibodies anti-rEhBvac21 located the EhV-ATPase B polypeptide in many small vesicles (Fig. 4a, c, g). The EhV-ATPase B subunit was also observed in EhkOs, but not in nuclei (Fig. 4a, c, and 4g, arrows). Some cells presented the EhV-ATPase B subunit in two EhkOs located in different planes (Fig. 4a, c, arrows). EhkOs were also stained with propidium iodide (PI) (Fig. 4b, d, and 4h, arrows) indicating they are DNA-containing organelles [15]. Nuclei did not appear PI stained in figure 4 because they were located in different planes (not shown) to those containing EhkOs. Colocalization of green and red fluorescent signals is also shown (Fig. 4e, i, arrows), corroborating that DNA and EhV-ATPase B subunit are in the same organelles. As a negative control we incubated cells without primary antibodies (Fig. 4k). Light microscopy images showed cellular integrity (Fig. 4f, j, and 4l). Immunolocalization of EhV-ATPase B subunit in EhkOs was confirmed by Western blot of an EhkO-enriched fraction prepared as described, which detected the 55 kDa band found in total protein extracts (Fig. 3d, e, lanes 3). We also determined the V-ATPase activity in this fraction (that was kept at -70°C) using the method described in [16], obtaining a specific activity of 6.04 nanomoles of phosphate produced in one hour per microgram of protein at 37°C.

### The *E. histolytica *vacuolar ATPase subunit B was found widely distributed in all cellular membranes in phagocytic trophozoites

**Figure 5:**
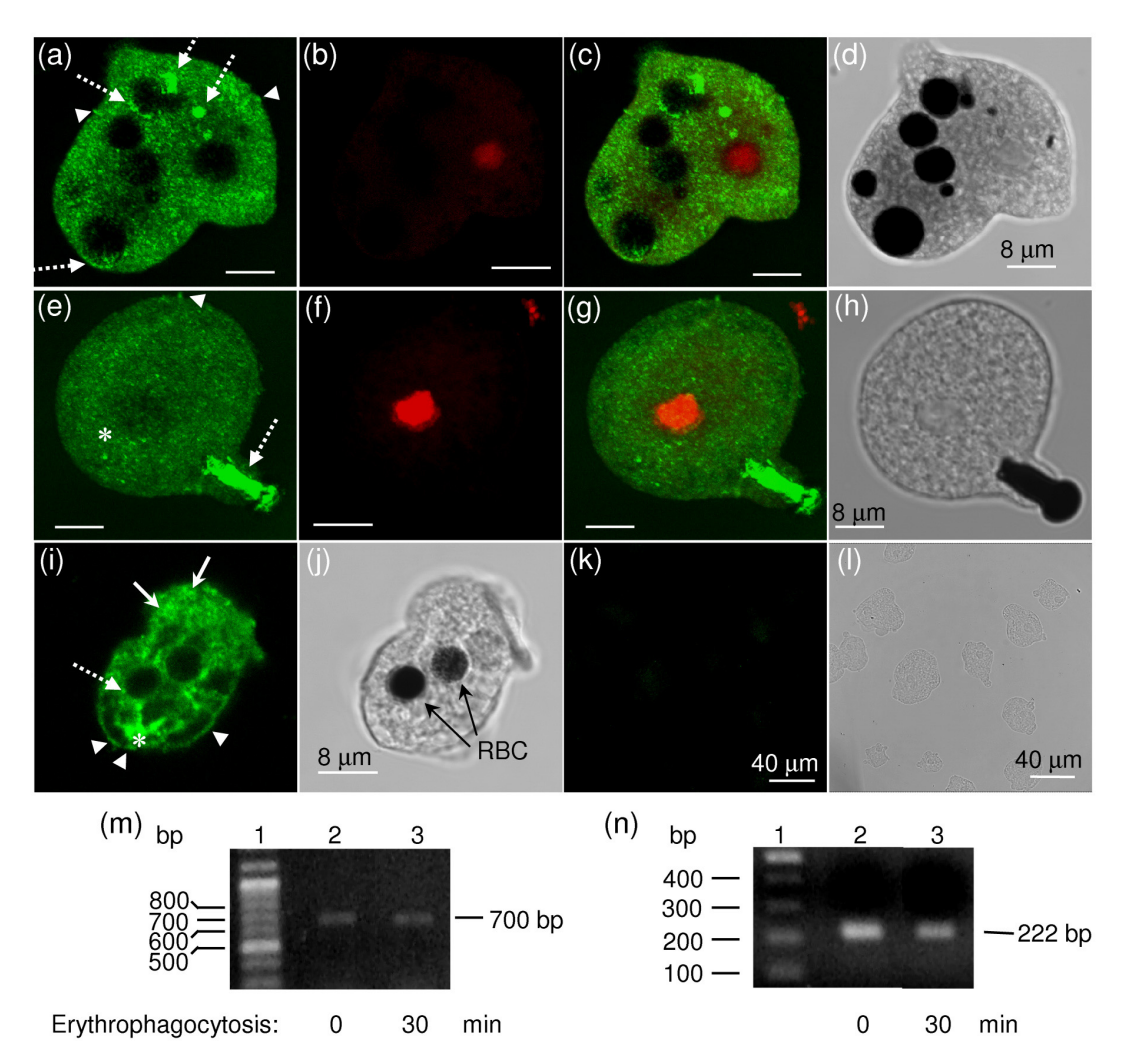
**Localization of EhV-ATPase B subunit in trophozoites during RBC phagocytosis**. Trophozoites were incubated with diamine bencidine-stained RBCs for 5 min (a-j), washed with PBS and fixed with paraformaldehyde. Then, cells were treated with anti-rEhBvac21 antibodies and FITC-labeled goat anti-mouse antibodies, and observed through a laser confocal microscope as described. (a, e, i). Immunodetection of EhV-ATPaseB observed in green channel. (b, f) Cells observed in red channel. (c, g) Merge of fluorescent signals observed in (a, b) and (e, f), respectively. (d, h, j, l) Cells observed by light microscopy. (k) Cells incubated only with secondary antibodies as negative control and observed in green channel. (m, n) Electrophoresis in 1.0% agarose gels of RT-PCR gene products of *Ehvma2 *(m) and actin (n) in trophozoites at 0 min (lane 2) and 30 min (lane 3) of erythrophagocytosis. Lane 1, 100 bp size molecular ladder. Arrowheads, EhV-ATPaseB found in plasma membranes. Arrows, EhV-ATPaseB polypeptide found in cytoplasmic vesicles. Dotted arrows, EhV-ATPaseB polypeptide found in phagocytic vacuoles containing RBCs. Asterisks, huge concentrations of EhV-ATPaseB found within trophozoites.

In confocal microscopy experiments of trophozoites during the phagocytosis of red blood cells (RBCs) we observed a wide distribution of EhV-ATPaseB protein through all the trophozoite membranes with the exception of nuclear membrane (Fig. 5). EhV-ATPaseB was found surrounding many vesicles of different sizes (Fig. 5i, arrows) and in plasma membrane (Fig. 5a, e, i, arrowheads). Some areas concentrated huge amounts of the protein located in cytoplasm (Fig. 5i, asterisk). The subunit B of EhV-ATPase was detected in phagocytic vesicles surrounding RBCs as expected (Fig. 5a, e, i, dotted arrows), confirming the presence of subunit B in phagocytic vacuoles as it has been previously reported [11]. However, our results showed no difference in the distribution of the subunit B of the vacuolar ATPase at the different times of phagocytosis tested. A negative control using preimmune serum was also carried out with no fluorescent signal (Fig. 5k). Nuclei were stained in red (Fig. 5b, f), and a merge of red and green fluorescent signals was also performed (Fig. 5c, g). It is noteworthy that subunit B was accumulated in huge amounts in the phagocytic mouth of a trophozoite ingesting a red blood cell (Fig. 5e). To determine if the *Ehvma2 *gene was being expressed in trophozoites during erythrophagocytosis, we carried out RT-PCR experiments that showed that this gene is being expressed (Fig. 5m). As control we used the actin gene that showed that it is also being expressed during this process (Fig. 5n).

### Putative subunits of the vacuolar ATPase found in the *E. histolytica *genome

We searched for the orthologous genes encoding for all the subunits of vacuolar ATPase in the *E. histolytica *genome database [17]. A BLAST search was done using the genes encoding for the vacuolar ATPase polypeptides from *Saccharomyces cerevisiae*. *E. histolytica *has orthologous genes encoding for all subunits of V_1 _complex of the vacuolar ATPase (Table 1). The corresponding *Entamoeba *genes were designated by the nomenclature used for the yeast vacuolar ATPase. *Ehvma1 *gene (Q24802 entry) encoded for the subunit A. Subunit B is encoded by *Ehvma2 *gene (Q4VSM4). We also found a pseudogene related to *Ehvma2*, denoted as Ψ *Ehvma2*, that was located at a different scaffold. Subunits C, F, G and H are encoded by *Ehvma5*, *Ehvma7*, *Ehvma10*, and *Ehvma13 *genes, respectively. On the other hand, we found two genes (*Ehvma8-1 *and *Ehvma8-2*) encoding for the putative subunit D located in different scaffolds, which predict proteins sharing a 98% identity. Subunit E is also encoded by two genes, *Ehvma4-1 *and *Ehvma4-2*, which are arranged in tandem on the same DNA strand in the *E. histolytica *genome (scaffold 9), and encode for proteins with a 51% identity between them. Orthologous genes encoding for the probable subunits of V_0 _complex were also found in the *E. histolytica *genome with the exception of c' and e subunits. V-ATPase a subunit is encoded by two genes, *Ehstv1 *and *Ehvph1*. The former is encoded at a single locus. The latter has two identical copies located at two different scaffolds. *Ehstv1 *and *Ehvph1 *predicted for putative proteins with 51% identity. Subunits c and c" are putatively encoded by *Ehvma3 *(Q24810 entry) and *Ehvma16*, respectively, and their polypeptides have 25% identity. A Blast search in the *E. histolytica *genome using the *S. cerevisiae *c' protein sequence (P32842 entry) as query reported the same genes mentioned above, but showing a lesser identity than that shown when comparing with *S. cerevisiae *subunits c and c" (data not shown), suggesting that *E. histolytica *genome lacks an orthologous c' subunit encoding gene. Finally, subunit d is encoded by a single copy of the *Ehvma6 *gene. Noteworthy all the V-ATPase subunit encoding genes are located in different scaffolds.

**Table 1:** Genes encoding for putative subunits of the vacuolar ATPase of *Entamoeba histolytica*

Subunit	Gene	Locus	Function^#^
A (Q24802)*	*Ehvma1*	EHI_043010^¶^	Catalytic site and regulation
B (Q4VSM4)*	*Ehvma2*	EHI_189850^¶^	Non-catalytic site
	ψ*Ehvma2*	122.t00032^§^	Involved in targeting?
C	*Ehvma5*	EHI_059840^¶^	Assembly and activity
D	*Ehvma8-1*	EHI_056390^¶^	Assembly and activity
	*Ehvma8-2*	202.t00016^§^	
E	*Ehvma4-1*	9.t00012^§^	Assembly and activity
	*Ehvma4-2*	9.t00013^§^	
F	*Ehvma7*	EHI_007940^¶^	Assembly and activity
G	*Ehvma10*	94.t00029^§^	Assembly and activity
H	*Ehvma13*	215.t00004^§^	Activity, but not assembly
a	*Ehvph1*	EHI_074020^¶^ 417.t00001^§^	H+ transport, assembly, and targeting
	*Ehstv1*	EHI_107280^¶^	
c (Q24810)*	*Ehvma3*	EHI_059840^¶^	H+-transport; DCCD-binding site
c"	*Ehvma16*	289.t00003^§^	H+ transport
d	*Ehvma6*	161.t00001^§^	Activity and assembly

*UniProtKB/TrEMBL Accession number.

^¶^Locus name assigned at Pathema (Bioinformatics Resource Center; ).

^§^Locus with its provisional names in the *E. histolytica *genome project at The J. Craig Venter Institute.

^#^Function was assigned based on reference [5].

## Discussion

The *Ehvma2 *gene encoding for the B subunit of V-ATPase is highly conserved (Fig. 1c). The protein was located in cytoplasm (showing punctate and diffuse patterns), plasma membrane, vacuolar membrane and in EhkOs (Fig. 4 and Fig. 5). Labeling in cytoplasm could correspond to free V_1 _sectors and to V-ATPase complexes found in small fluorescent vesicles which might be lysosomes [18,19]. In yeast cells, the RAVE complex mediates the reversible dissociation between the V_1 _and V_0 _complexes by binding to peripheral stalk subunits (C or E and G) [20,21], that is considered as one of the mechanisms controlling V-ATPase activity [20-22]. This event seems to be regulated by the cellular environment as in yeast in response to glucose depletion [23]. The gene encoding for the Skp1 subunit of the RAVE complex is found in the *E. histolytica *genome (there are four gene sequences encoding for this subunit in Pathema: EHI_118670, EHI_174130, EHI_066770 and EHI_174180), suggesting a similar V-ATPase regulation mechanism in this parasite.

On the other hand, the V-ATPase B subunit found in the *E. histolytica *plasma membrane (Fig. 5) could indicate that the V-ATPase complex is located there and has a role in tissue invasion in a similar process of metastasizing cells that increase the acidification of the extracellular fluid to produce the destruction of normal tissue and facilitate invasion [3]. Moreover, it could be involved in the transport of diverse solutes in a similar way as it has been described for *Plasmodium *[24]. It is noteworthy the subunit B localization in EhkOs (Fig. 4), which are cytoplasmic DNA-containing organelles of unknown function [15,25]. EhkOs carry EhTBP [26], EhC/EBP [27], and Ehp53 [28] transcription factors, and the EhPFO enzyme [29], but their roles in EhkOs remain to be determined. Another cytoplasmic DNA-containing organelle described in this parasite is the crypton, which harbors the chaperonin Hsp60 [19,30].

Mitosomes, which are DNA-lacking organelles, also contain the chaperonin Cpn60 and are considered as mitochondrial remnants [31]. Mitosomes have also been found in other organisms [32-34]. EhkOs and cryptons could be similar organelles as they carry DNA. However, EhkOs and mitosomes are clearly different [35]. A possible role for the V-ATPase located in EhkOs could be the ATP synthesis using the membrane-bound H^+^- translocating inorganic pyrophosphatase (H^+^-PPase) activity found in some organelles in trophozoites [36]. Alternatively, V-ATPase could be pumping out H^+ ^as a consequence of the B subunit localization towards the lumen of these organelles (Fig. 4), in order to keep pH regulated and avoid degradation of their content.

RBCs ingestion by trophozoites revealed a clustering process of EhV-ATPase B subunit in the vicinity of phagosomes after 5 min of phagocytosis (Fig. 5). The principal route of V-ATPase delivery to phagosomes in *D. discoideum *is by fusion with pre-existing acidic endosomal vesicles, prior to their content delivery into phagosomes [37]. A similar process might be occurring in *Entamoeba*, where lysosomes could be stored in the cytosol prior to their fusion with phagosomal vacuoles. During erythrophagocytosis a pre-phagosomal vacuole (PPV) containing Rab5 and Rab7A proteins is gradually acidified by fusion with lysosomes. During the first 5–10 min Rab5 is dissociated from PPVs, and digestive proteins are transported from PPVs to phagosomes via Rab7A-mediated vesicular transport [38,39]. Although this process needs more studies to identify all proteins involved, V-ATPase should be released during PPV maturation. In addition, interactions of V-ATPase to cytoskeleton through subunits B and C, which are able to bind actin, are essentials for its proper function. For example, the osteoclast subunit B is responsible for microfilaments-V-ATPase interactions [40]. The putative actin-binding domain that was found in EhV-ATPase subunit B suggests that this process is conserved in this parasite (Fig. 1).

**Figure 6:**
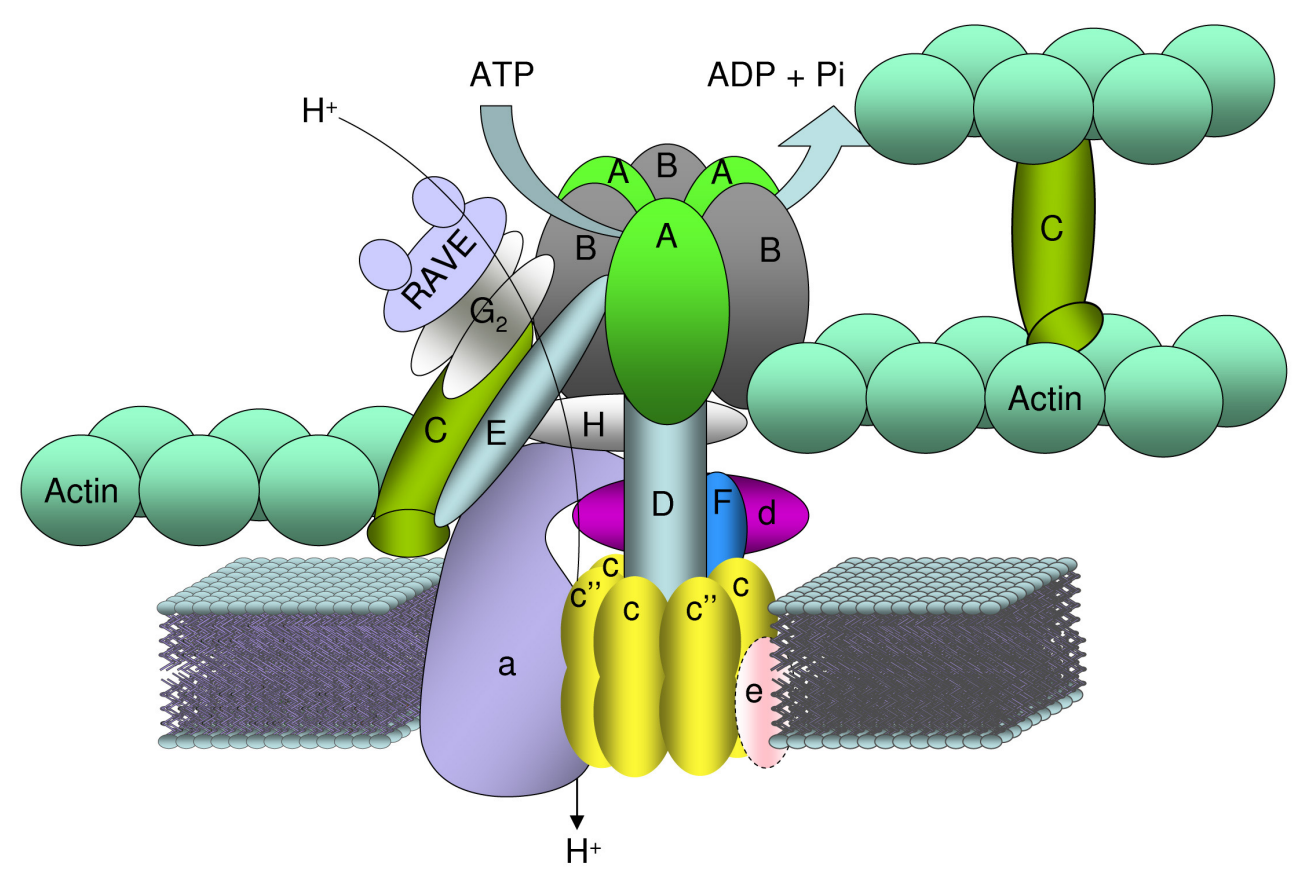
**Hypothetical model of the structure of the vacuolar ATPase of Entamoeba histolytica**. The Vacuolar ATPase of *E. histolytica *is a well conserved protein complex, with the exceptions of subunits c' and e, which have not conserved counterparts in this parasite. The structure shown is according to that described in [3,5] and based on data from Table 1. Subunit e that was not found in the *E. histolytica *genome is surrounded by a dotted line.

In eukaryotic cells the V_1 _complex is formed by the subunits A_3_B_3_CDEFG_2_H_1–2 _and the V_0 _complex by subunits ac_4_c'c"de [3-5]. Based on the genes found in the *E. histolytica *genome, we propose a putative model for the structure of the vacuolar ATPase in this parasite (Fig. 6). The V_0 _complex might be composed of a ring of five or six proteolipid subunits (four c subunits and either one or two c" subunits, respectively) and one molecule of each a and d subunits. Remarkably, *E. histolytica *V-ATPase lacks subunits e and c'. However, the existence of non-related proteins to e and c' subunits that could evolve separately very early in evolution in *Entamoeba *is possible. It is important to note that the stoichiometry of the E and G strong interaction is not well established yet. A relationship of two G molecules per one E molecule was assumed, however recent data support that in V-ATPase, G and E subunits are present in more than one copy and thus two peripheral stalks connecting V_1 _to V_0 _could exist [41].

Most of the subunits of the *E. histolytica *V-ATPase are encoded by single genes, excepting for subunits D, E, and a, which are encoded by two genes in each case (Table 1). In the case of the subunit E, two E isoforms have been found in mouse [6], and three in *Arabidopsis *[42]. Several pea epicotyls V-ATPases isoenzymes exhibiting different ratios of E1 and E2 isoforms have been purified, exhibiting differences in their Km and Vmax of ATP hydrolysis, and different tissue distribution in the whole plant [43]. Yeast has two subunit a isoforms: 1) Vph1p, which is targeted to the vacuolar membrane, and 2) Stv1p which is targeted to the late Golgi apparatus [44]. Mammals have four genes encoding for different subunit a isoforms, often showing cell-type or tissue-specific locations [5]. A special case is *Paramecium*, which contains 17 a subunit isoforms, which provide a specific location and function for V-ATPase in at least seven different compartments [45]. Thus, *E. histolytica *could have at least two V-ATPase isoforms, one distributed in vacuoles, and the other in the Golgi apparatus that could be more primitive in trophozoites than those from other systems due to the lack of a typical Golgi structure [46-48]. More studies are needed to elucidate the V-ATPase role in the physiology of *E. histolytica *trophozoites.

## Conclusion

We cloned the *Ehvma2 *gene which encodes for the highly conserved V-ATPase B subunit. This protein was immunolocated in EhkOs, in plasma membrane, vesicles, phagocytic vacuoles, and also in the cytoplasm. Most of the orthologous genes encoding for the V-ATPase subunits were also identified in the *E. histolytica *genome showing that this EhV-ATPase is a highly conserved complex. The *in silico *analysis of the genes encoding for the V-ATPase subunits in the *E. histolytica *genome revealed that the V_1 _complex has all the eight subunits found in V-ATPases. However, the V_0 _complex lacks the subunits e and c'.

## Methods

### *E. histolytica *cultures

Trophozoites of *E. histolytica *clone A were axenically cultured in TYI-S-33 medium at 37°C and harvested during the exponential growth phase as described [49]. For EhkOs purification, the medium was supplemented with 2 μCi/ml [methyl-^3^H]-Thymidine (Amersham) for 48 h [50].

### Search of V-ATPase genes in the *E. histolytica *genome database

A search of V-ATPase genes was performed in the *E. histolytica *genome databases at The Sanger Institute  and The J. Craig Venter Institute . As query, we used the subunit polypeptide sequences of the vacuolar ATPase of *S. cerevisiae*, and the WU-BLAST version 2.0 program and BLOSUM62 matrix. The UniProt Knowledgebase (UniProtKB)/TrEMBL entries used were P17255 (subunit A), P16140 (subunit B), P31412 (subunit C), P32610 (subunit D), P22203 (subunit E), P39111 (subunit F), P48836 (subunit G), P41807 (subunit H), P37296 (subunit a), P25515 (subunit c), P32842 (subunit c'), P23968 (subunit c"), P32366 (subunit d), and Q3E7B6 (subunit e). Obtained DNA sequences were translated to proteins with the Translate tool at the ExPASy Proteomics Server . Blast search for each of the *Entamoeba *polypeptide sequences was done with BLASTP 2.2.14 algorithm in The UniProt Knowledgebase at the ExPASy Proteomics server of the Swiss Institute of Bioinformatics and BLOSUM62 matrix. Alignments were performed with ClustalW version 1.83 algorithm at the European Bioinformatics Institute (EBI, ).

### Cloning of the *Ehvma2 *gene encoding for the subunit B of the *E. histolytica *vacuolar ATPase

Based on the nucleotide sequence of the putative *Ehvma2 *gene found in the *E. histolytica *genome databases, we designed the primers S-Bvac (sense): 5'-ATGTTATTGTATTAAGACTTTTTAATT-3', and AS-Bvac (antisense): 5'-TATAAGGTTCCTTCTTTGTCGT-3' to amplify a 1.87 kb DNA fragment using 50 ng of total DNA as a template, and 400 μM of each dNTP, 2.5 mM MgCl_2_, 300 nM of each primer and 2 U of *Taq *DNA polymerase (Invitrogen). PCR conditions were 94°C for 5 min, 30 cycles of 94°C for 30 s, 57°C for 30 s and 72°C for 90 s, and 72°C for 10 min. Amplified DNA was cloned into the pGEM-T-Easy vector (Promega) to generate pGEM-T-Easy-Bvac plasmid. Sequencing of cloned DNA was carried out using gene specific primers and the Big Dye Terminator kit version 2.0 in an Automated DNA Sequencer (310 Genetic Analyzer, Applied Biosystems) in the Sequencing Nucleic Acid Core Facility at Cinvestav-IPN.

### RT-PCR

Total RNA was isolated from trophozoites using TRIzol reagent (Invitrogen) and reverse transcription was carried out with 200 U of SuperScript II reverse transcriptase (Invitrogen) and 40 U of SUPERase-in RNAse inhibitor (Ambion) according to manufacturers' instructions and 1/6 of reaction volume was used for DNA amplification by PCR with Bvac-S-1 (5'-ATGGAAGCTTTCAAAATT-3') sense and Bvac-AS-833 (5'-CTTTCCATAGAACCATTTTC-3') antisense primers using the PCR conditions described above. RT-PCR products were separated on a 4% PAGE gel in 1× TBE (90 mM Tris, 90 mM H_3_BO_3_, and 2 mM EDTA, pH 8.3), stained with 0.05% (w/v) ethidium bromide (EtBr), visualized with a UV transilluminator, and images were processed with a Gel Documentation System (Bio-Rad). For RT-PCR analysis of *Ehmav2 *and *actin *genes in trophozoites during RBCs erythrophagocytosis, we proceeded as described bellow, but no fixative agents were employed. For *actin *gene we used primers actin-sense 5'-AGCTGTTCTTTCATTATATGC -3' and actin-antisense 5'-TCTCTTTCAGCACTAGTGGT-3', and used a Tm of 54°C.

### Expression and purification of a recombinant fragment polypeptide of *E. histolytica *V-ATPase subunit B in *Escherichia coli*

To express a specific region of EhV-ATPaseB, we amplified by PCR (using the conditions mentioned above) a 478 bp DNA fragment (containing a 465 bp DNA encoding for a 154 aa polypeptide of EhV-ATPaseB and the translation stop signal). The primers used were Bvac-S-373 (5'-CGGGATCCGGTATTGATGCACGTTATAC-3') sense and Bvac-AS-833 (5'-CCCAAGCTTTTATCTTTCATAGAACCATTTTC-3') antisense, which contained the *Bam*HI and *Hind*III restriction sites at their 5' ends, respectively. Then, the amplified DNA was cloned into pRSET A vector (Invitrogen) to generate the recombinant pRSET-EhBvac465 plasmid. Recombinant protein expression was induced with 1 mM IPTG in *E. coli *BL21(DE3) pLysS (Invitrogen). N-terminal His-tagged recombinant EhBvac 21 kDa (rEhBvac21) polypeptide was purified under denaturing conditions by IMAC using a Ni^2+^-NTA agarose column (Qiagen), following the manufacturer's protocol. Purification of rEhBvac21 polypeptide was improved by electroelution from preparative 15% SDS-PAGE gels.

### Generation of mouse polyclonal antibodies against the rEhBvac21 polypeptide and Western blot assays

Specific antibodies against EhV-ATPaseB were generated in BALB/c mice by immunization of 100 μg of purified rEhBvac21 polypeptide mixed with Freund's adjuvant. Three bursts were applied via intraperitoneal at ten day intervals. Immune serum was collected seven days after the last immunization and used for Western blot assays. Mice were bleeding before immunization to use pre-immune sera as a control in all experiments. For Western blot assays total protein extracts of induced bacteria or purified rEhBvac21 polypeptide were separated by 15% SDS-PAGE, transferred to nitrocellulose membranes, and blocked. This was followed by incubation with mouse anti-His monoclonal antibodies (0.3 μg/ml) (Roche) for 1 h at 37°C, or by incubation with mouse polyclonal anti-rEhBvac21 antibodies (1:1,000) overnight at 4°C. Then, membranes were washed and incubated with horseradish peroxidase-conjugated anti-mouse IgG secondary antibodies (Zymed) (1:1,000) for 3 h at 37°C. Immunoreactive bands were revealed with 4-chloro-1-naphthol and 0.03% (v/v) H_2_O_2_. For Western blot analysis of total trophozoite extracts and EhkO-enriched fraction, proteins were separated on 10% SDS-PAGE. Then, membranes were incubated with anti-rEhBvac21 antibodies (1:20,000) for 3 h at 37°C, and secondary antibodies (1:3,000) at 37°C for 1 h. Positive bands were detected using the ECL Plus detection kit (Amersham) followed by exposure to X-ray films (Kodak).

### Immunofluorescence and confocal microscopy

Trophozoites on prewarmed cover slips were fixed with 3% (w/v) paraformaldehyde for 1 h at 37°C, incubated with 50 mM NH_4_Cl for 1 h at 37°C, permeabilized with 0.5% (v/v) Triton X-100 at 37°C for 30 min and blocked with 0.1% (w/v) BSA in PBS at 37°C for 30 min. Then, cells were incubated with mouse polyclonal anti-rEhBvac21 antibodies (1:80) for 2 h. Subsequently, cells were washed with PBS and incubated with FITC-labeled goat anti-mouse secondary antibodies (1:200) in blocking solution at 37°C for 2 h. Finally, cells were washed and counterstained with 20 μg/ml PI (Fluka), and observed through a Leika NT-TCS confocal microscope. As a control, cells were incubated without the anti-rEhBvac21 antibodies.

### Phagocytosis assays

Trophozoites were incubated with human RBCs at 37°C for different times (2 to 120 min) as described [51]. At the end of the incubation times, the culture medium was removed and trophozoites were washed with PBS and fixed with 3% (w/v) paraformaldehyde. For immunofluorescence experiments cells were treated as described before.

### Isolation of an EhkO-enriched fraction and V-ATPase activity assay

EhkO-enriched fraction purification steps were carried out at 4°C as described [50]. [^3^H]-Thymidine labeled trophozoites were washed and resuspended in eight volumes of buffer A (10 mM HEPES pH 7.9, 10 mM EDTA, and 10 mM DTT) containing protease inhibitors and 250 mM sucrose. Cells were gently disrupted on ice using a Potter homogenizer and centrifuged at 160 × g for 10 min. Then, the supernatant (nuclei-depleted fraction) was centrifuged at 10,000 × g for 10 min at 4°C in a JA-20 rotor (Beckman) to obtain the soluble fraction and the pellet. The latter was resuspended in 15% (v/v) Nycodenz (Nycomed Pharma AS) in buffer A and top loaded on a Nycodenz discontinuous gradient (30, 40 and 50%, v/v), which was centrifuged at 13,000 × g for 60 min at 4°C in a SW40Ti rotor (Beckman). 0.5 ml fractions were collected with a DensiFlow II C system (Buchler Instruments) and a RediFrac 1,000 fraction collector (Bio-Rad). 100 μl of each fraction were 10% (w/v) TCA precipitated and radioactivity content was determined in a LS6500 liquid scintillation counter (Beckman). EhkO enriched fractions were identified by its [^3^H]-Thymidine incorporation.

The vacuolar ATPase activity was determined using a modification of the method described by Conibear and Stevens [16]. The sample was incubated in 50 mM MES/Tris buffer pH 6.9, 5 mM MgSO_4 _and 5 mM ATP at 37°C for 16 h. Some reactions were performed incubating some samples with 0.1 mM Na_3_VO_4_, or 300 nM concanamycin A, or both. Released phosphate was determined by absorbance at 630 nm after a 15 min incubation period at RT with a mixture of 1% (w/v) (NH_4_)_6_Mo_7_O_24_, 4% (w/v) H_2_SO_4_, 1% (w/v) SDS and 0.2% (w/v) ascorbic acid.

## Authors' contributions

All authors conceived the study. MGMH isolated and characterized the *Ehvm2 *gene, expressed the recombinant polypeptide in bacteria, obtained the anti-EhV-ATPase subunit B antibodies, purified EhkOs and performed the confocal microscopy studies. MLLB helped in RT-PCR assays and measurement of vacuolar ATPase activity. MGMH and JPLA performed the *in silico *analysis. EO and JPLA drafted the manuscript. All authors read and approved the final manuscript.
